# Combined revision ACL reconstruction with slope‐correction osteotomy and lateral extra‐articular tenodesis improves stability in patients with high posterior tibial slope and pivot shift

**DOI:** 10.1002/jeo2.70384

**Published:** 2025-07-24

**Authors:** Jesper Fritz, Alan Getgood, Ronald van Heerwaarden, Sebastien Parratte, Charles Brown, Luke V. Tollefson, Robert F. LaPrade

**Affiliations:** ^1^ International Knee and Joint Centre Abu Dhabi UAE; ^2^ Fowler Kennedy Sports Medicine Clinic London Ontario Canada; ^3^ Centre for Deformity Correction and Joint Preserving Surgery, Kliniek ViaSana Mill the Netherlands; ^4^ Twin City Orthopedics Edina Minnesota USA

**Keywords:** high‐grade anterior knee instability, increased posterior tibial slope, revision anterior cruciate ligament reconstruction, slope‐correction osteotomy

## Abstract

**Purpose:**

The purpose of this study was to evaluate the outcomes of patients undergoing single‐stage revision anterior cruciate ligament (ACL) reconstruction (ACLR) with bone‐patellar tendon‐bone (BPTB) autograft, anterior closing wedge proximal tibial osteotomy (ACWPTO) and lateral extra‐articular tenodesis (LET).

**Methods:**

An institutional review board‐approved retrospective study of all patients who underwent a revision ACLR using a BPTB autograft, ACWPTO and LET from a single centre from 2018 to 2023 was performed. Inclusion criteria were patients >18 years of age with a failed ACLR, posterior tibial slope (PTS) of >15°, previous ACL‐tunnel diameters of <14 mm, and intact ipsilateral patellar tendon. PTS and anterior tibial translation (ATT) were measured using the mechanical axis on long weight‐bearing lateral tibial radiographs.

**Results:**

Nine patients, all men, were evaluated with a mean age of 31.1 years and a mean follow‐up of 31.4 months. The PTS significantly decreased from 16.8° (range: 15.1°–18.9°) preoperatively to 9.3° (range: 5.0°–14.7°) post‐operatively (*p* < 0.001) and ATT significantly decreased from 14.6 mm (range: 10.7–19.0 mm) preoperatively to 6.3 mm (range: 1.3–11.5 mm) post‐operatively (*p *< 0.001). Preoperatively, all patients showed significant instability with the Lachman test Grade 2/3 and the pivot shift test Grade 2/3. Post‐operatively, Lachman test grade was 0 and Pivot shift test grade was 0 in all patients (*p* < 0.01), and the average post‐operative subjective International Knee Documentation Committee (IKDC) score was 79.4 (range: 60.9–95.4).

**Conclusions:**

Single‐stage revision ACLR using BPTB autograft, ACWPTO and LET in an ACL‐deficient knee with high‐grade pivot shift and increased PTS was safe and reliable, with significantly improved clinical and objective outcomes.

**Level of Evidence:**

Level IV, case series.

**Abbreviations**
ACL: anterior cruciate ligament
ACLR: anterior cruciate ligament reconstruction
ACWPTO: anterior closing wedge proximal tibial osteotomy
ATT: anterior tibial translation
BPTB: bone‐patellar tendon‐bone
FCL: fibular collateral ligament
IKDC: Internation Knee Documentation Committee
ITB: iliotibial band
LET: lateral extra‐articular tenodesis
PTS: posterior tibial slope
ROM: range of motion

## BACKGROUND

Even though modern techniques for anterior cruciate ligament (ACL) reconstruction (ACLR) demonstrate excellent results, there is a reported failure rate of 7%–20% [3, 6, 26, 28]. Evaluation of ACLR failure is multifactorial, but high‐grade anterior knee laxity and increased posterior tibial slope (PTS) have been reported to be independent risk factors [14, 32].

Increased PTS results in a higher load on the ACL [1], and increased overall anterior tibial translation (ATT) and thereby presents an increased risk of ACLR graft failure [2, 4, 17]. Middle eastern patient populations are especially susceptible to this due to their increased normal average PTS of 13.6° compared to a lower average PTS in other populations [37]. Another factor increasing the ATT, as well as the pivot shift and internal rotation, is insufficiency of the anterolateral structures. Addressing these risk factors during surgery is valid [12], because anterior closing wedge proximal tibial osteotomy (ACWPTO) reduces the load on the ACL [17] and a lateral extra‐articular tenodesis (LET) has reported to reduce the anterolateral laxity, pivot shift and possibly reduce the risk of ACLR failure and revision ACLR failure in patients with a high grade pivot shift (Grades 2 and 3) [13].

Weighing the pros and cons and making an individualized graft choice is the modern approach to ACLR. In patients with a high‐grade pivot shift grade and increased PTS, the main characteristic for graft choice should be a low failure rate. Even though it has been debated, large recent studies suggest the bone‐patellar tendon‐bone (BPTB) autograft to have the lowest failure rate [21, 29]. Due to technical difficulty for a BPTB autograft harvest combined with a concurrent ACWPTO, this has been disregarded in the past. Instead, previous solutions for the failed ACLR with high‐grade pivot shift and increased PTS include two‐stage procedures [1, 33] or using other grafts [8, 9, 27, 31, 35]. However, a single‐stage surgery for a failed ACLR with high tibial slope would be beneficial because it decreases the number of surgeries, the rehabilitation time, and overall costs.

With modern techniques for a BPTB graft harvest, LET and ACWPTO, we believe the technical difficulties considered in the past are no longer valid to avoid this combination. The systematic review by Tollefson et al. [34] reported on outcomes for slope‐reducing osteotomies with or without concomitant ACLR; however, no studies in this systematic review reported specifically on single‐stage procedures with the addition of an LET [34]. We hypothesized that a single‐stage revision ACLR with a BPTB autograft, ACWPTO and LET was safe and improved clinical and patient‐reported outcomes.

## METHODS

This was a retrospective study of all patients undergoing a revision ACLR using a BPTB autograft, ACWPTO and LET from 2018 to 2023 by two different board‐certified sports medicine surgeons (JF, CB) at a single centre (International Knee and Joint Centre). It has been reported that a PTS of >12° places an ACLR graft at a higher risk of failure. To reduce the risk of unnecessary osteotomies, an inclusion criterion of PTS > 15° was chosen, similar to other studies [34]. Other inclusion criteria were patients who had provided research consent, were >18 years of age with a failed ACLR, PTS of >15°, previous ACL‐tunnel diameters of <14 mm, and anatomic reconstruction tunnel position. Exclusion criteria were concomitant other ligament reconstructions, previous BPTB graft harvest and previous LET. There were no dropouts during follow‐up. Approval from the institutional research review committee was obtained (MF3867‐2024‐1).

### Surgical technique

An examination under anaesthesia was performed. The LET site was approached first. A 4 cm lateral incision was made to approach the iliotibial band (ITB) where an approximately 10 mm wide and 10 cm long strip of the ITB was harvested from its posterior half, cut proximally and left intact distally to be used as the LET graft. The strip was passed deep to the fibular collateral ligament (FCL) from distal to proximal, following which the proximal lateral femoral attachment site for the LET (dorsally on the lateral femur in between the distal and proximal Kaplan fibre attachments) was marked and decorticated [19].

**Figure 1:**
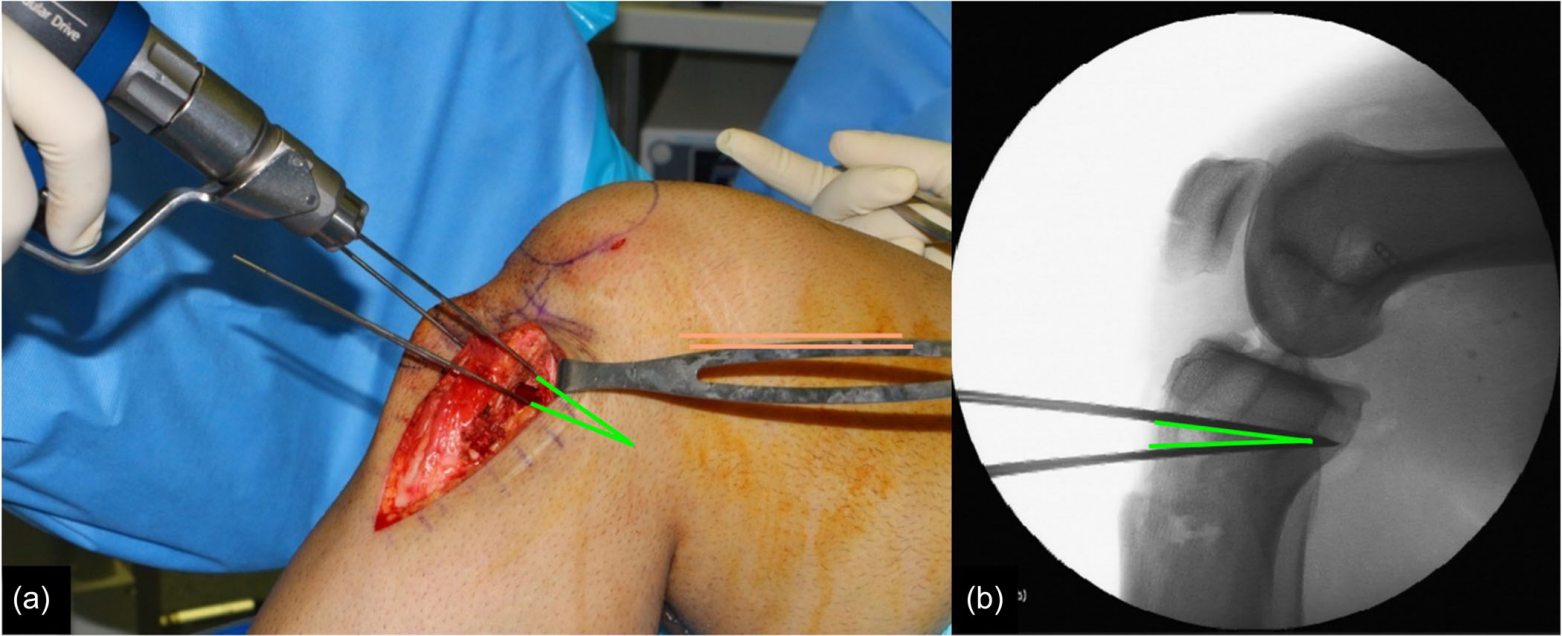
After the bone‐patellar tendon‐bone autograft harvest, two medial and two lateral pins are put in place to mark the osteotomy. Preoperative calculations reveal the size of the wedge, and the pins converge at the posterior aspect of the tibia. (a) An anteromedial image of the open incision. (b) A lateral fluoroscopic image is taken when the pins are inserted but before the osteotomy is performed. The green lines represent the osteotomy wedge that is to be removed.

**Figure 2:**
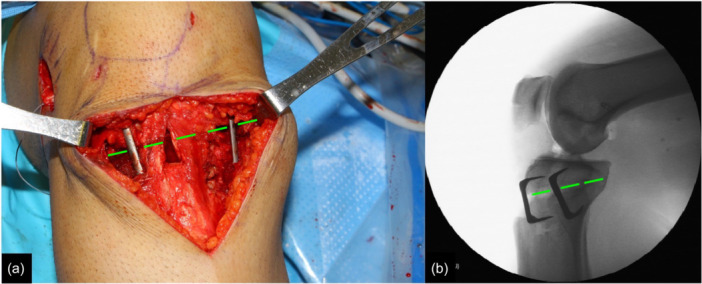
After the anterior osteotomy wedge has been resected and the osteotomy is closed in extension, one medial and one lateral staple are used for fixation. (a) An anterior image of the open incision. (b) Lateral fluoroscopic image. The dotted green lines represent the closed osteotomy site.

Next, the ACWPTO was performed, starting with an 8‐cm‐long anteromedial incision of the proximal tibia and soft tissue dissection medially and laterally using a Cobb elevator to elevate deep to the superficial medial collateral ligament, rounding the posteromedial corner and positioning a blunt Hohmann retractor for posterior protection and a retractor to protect the anterolateral compartment soft tissues. Under fluoroscopic control, two medial and two lateral guide pins were inserted for the planned osteotomy, avoiding Gerdy's tubercle (Figure 1a,b). A supratubercle osteotomy approach was taken, but depending on the planned osteotomy size, either simple anterior‐to‐posterior osteotomy cuts or a biplanar osteotomy to avoid the tibial tubercle was performed. Due to the increased normal PTS in Middle Eastern patients of 13.6°, the target post‐operative PTS was 7°–12° in order to avoid excessively flat tibial slope, which could lead to increases in knee hyperextension [37]. The osteotomy was closed in extension and fixed with one medial and one lateral staple (Figure 2a). Fluoroscopy was used to verify proper closure and fixation of the osteotomy (Figure 2b). Similar techniques have been described in the literature, but not all perform the osteotomy proximal to the tibial tubercle or use a BPTB graft [8, 15].

Using the same incision, a 1 cm wide BPTB graft was harvested from the patellar tendon with a 10 × 20 mm bone plug from the patella and a 10 × 25 mm bone plug from the tibia. The graft was prepared on the back table and soaked in vancomycin (5 mg/mL normal saline) to be used as a revision ACLR graft.

Arthroscopy was then performed, and cartilage lesions and meniscus tears were appropriately treated. Using standard guides, the ACL femoral and tibial tunnels were independently drilled in an outside‐in fashion, the tibial tunnel placed proximal to the osteotomy site, and thereafter the BPTB graft was inserted retrograde and fixed in 20° knee flexion with titanium interference screws in both the femur and tibia in a standard fashion.

Finally, the LET femoral fixation was performed when the graft was fixed to the femur with a staple in 60° of knee flexion and neutral tibial rotation, as this has been shown to reduce the risk of high contact pressure in the lateral compartment [18]. After staple fixation, the remaining graft was folded over the staple and sutured back to itself. All incisions were closed in a standard fashion.

### Post‐operative rehabilitation

The same post‐operative protocol was used for all patients. Weightbearing was restricted to flat foot touch for the first 4 weeks post‐operatively, 25%–50% of bodyweight from 4 to 6 weeks post‐operatively, 50%–75% of bodyweight from 6 to 8 weeks post‐operatively and full weightbearing as tolerated after 8 weeks. A similar increasing approach was taken for range of motion (ROM), with the first 4 post‐operative weeks allowing 0°–90° ROM, 4 to 6 weeks post‐operatively 0–120° ROM, 6 to 8 weeks post‐operatively 0°–135° and thereafter full ROM was allowed. Hyperextension is avoided to prevent increased post‐operative recurvatum from the ACWPTO.

### Radiographic measurements

**Figure 3:**
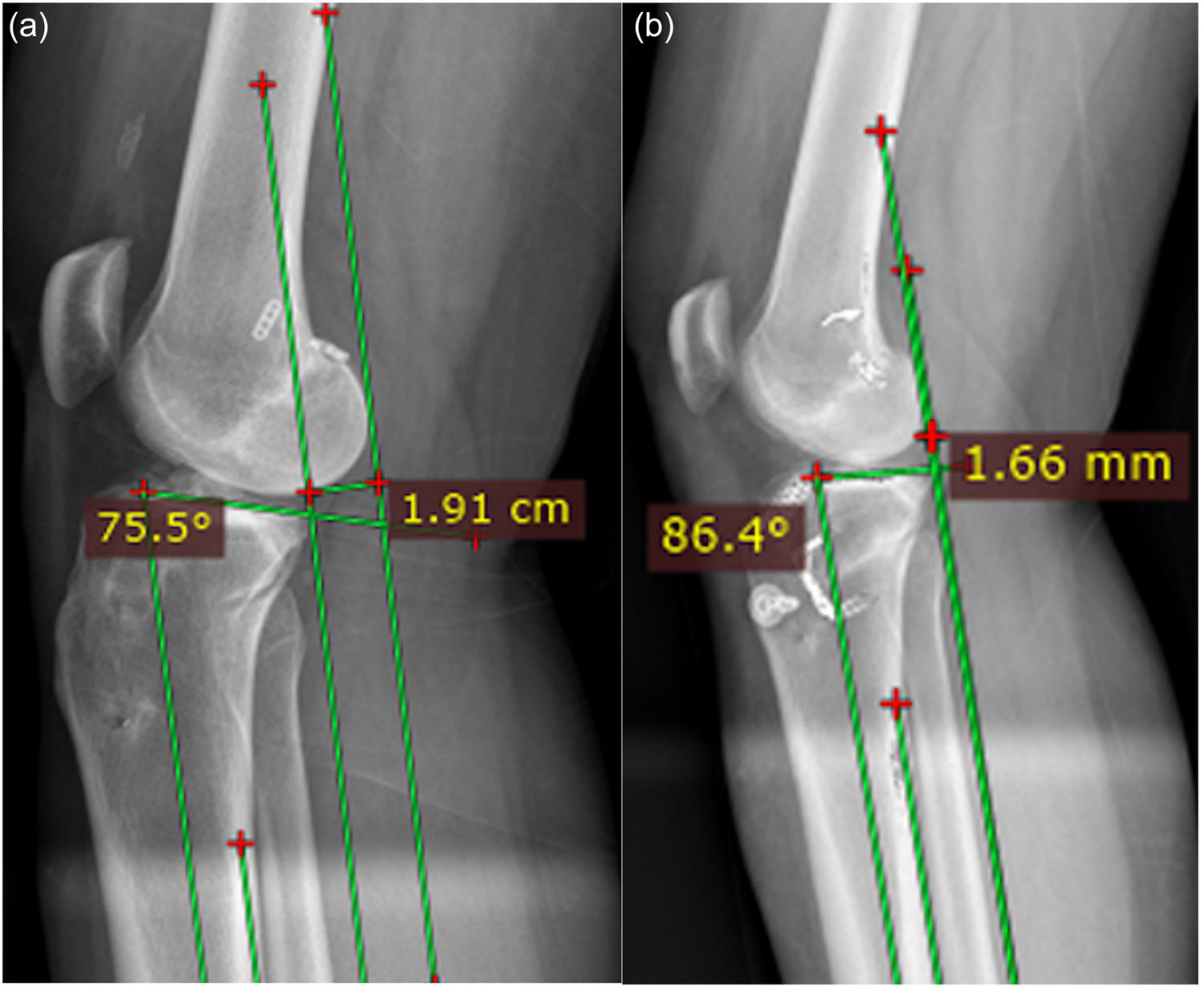
Preoperative (a) versus post‐operative (b) measurements of both posterior tibial slope (PTS) and anterior tibial translation (ATT). PTS was measured as the angle between the mechanical axis of the tibia and the medial tibial plateau. ATT was measured as the perpendicular distance between the posterior medial femoral condyle and medial tibial plateau using the posterior tibial cortex as a reference. In the preoperative image (a), the PTS is measured at 14.5° (90°–75.5°) and the ATT at 19.1 mm. In the post‐operative image (b), the PTS is measured at 3.6° (90°–86.4°) and the ATT at 1.66 mm.

Standard long weightbearing lateral radiographs in 20°–30° of flexion (single leg monopodal stance view) were obtained prior to surgery and at least three months after surgery to calculate the ATT and PTS (Figure 3).

ATT was measured as the perpendicular distance between the most posterior aspect of the medial femoral condyle and the medial tibial plateau using lines corresponding to the axis of the posterior tibial cortex [10]. PTS was measured using the mechanical axis, defined as the line between the mid‐points from anterior to posterior 5 cm from the proximal and distal ends of the tibia and the medial tibial plateau, as previously extensively described [25, 30]. To reduce variability in measurements and to mitigate bias, two orthopaedic surgeons (JF, CB) measured PTS and ATT on all radiographs and the means were used for analysis.

### Clinical outcome

The Lachman, pivot shift and anterior drawer tests were all performed preoperatively and post‐operatively to evaluate knee stability by two different physicians (JF, CB). The Lachman test was performed in 20° of knee flexion, while pivot shift was initiated with a slight valgus and internal rotation force with the knee in full extension. To subjectively evaluate the functional outcomes, all patients completed the International Knee Documentation Committee (IKDC) subjective scores at their last follow‐up. Medical records and interviews were used to evaluate if the patient had any secondary surgery post‐operatively.

### Statistical analysis

For comparisons of PTS and ATT, a paired *t* test was used. For comparisons of the distribution of the clinical tests, the Mann–Whitney *U* test was used. Measurements of PTS and ATT were taken at two separate timepoint and the average between the time points was used for analysis. The intraclass correlation coefficient (ICC) was used to calculate the reliability of the measurements. Post hoc power analysis of changes is PTS and changes in ATT both revealed a power of 1.0, suggesting this study had sufficient power for statistical analysis. The Shapiro–Wilk test was used to test for normality. All the main data points rejected the null hypothesis of the data not being normally distributed, with *p* values of 0.941 and 0.511 for preoperative PTS and ATT and *p* values of 0.149 and 0.527 for post‐operative PTS and ATT. Excel (Microsoft) and SPSS v.28.0 (IBM) were used for analysis, and the statistical significance was defined as *p* < 0.05.

## RESULTS

Nine patients, all men; mean age, 31.1 years (range: 20.6–48.7 years, 95% confidence interval [CI] = 24.0–38.2), were evaluated, with a mean follow‐up of 31.4 months (range: 15.6–60.3 months). Eight patients had one previous ACLR, and one patient had two previous ACLRs. All patients had hamstring autografts for the previous ACLRs (Table 1). One patient had a 10° flexion deficit at final follow‐up; the rest had full ROM, compared to the contralateral side. The medial meniscus was partially resected in two cases and repaired in one case in an inside‐out fashion due to a radial tear in the posterior horn. The lateral meniscus was partially resected in two cases, repaired in one case in an inside‐out fashion due to a longitudinal tear in the posterior horn and a lateral meniscus root was repaired using a transtibial tunnel and two permanent sutures in two cases. There were two cases of cartilage injury grade III detected, in the trochlea and on the central portion of the patella, but no cartilage restoration procedure was performed.

**Table 1:** Patient age, height, weight and previous surgeries of the affected knee (n = numbers, graft used).

				Previous ACLR in the same knee	
Patient	Age (years)	Height (m)	Weight (kg)	*n*	Graft used
1	20.6	1.71	91	1	Ispilateral hamstring autograft
2	30.2	1.71	81	1	Ispilateral hamstring autograft
3	48.7	1.58	62	1	Ispilateral hamstring autograft
4	31.2	1.65	65	1	Ispilateral hamstring autograft
5	29.2	1.8	90	1	Ispilateral hamstring autograft
6	30.0	1.82	106	2	Ispilateral + contralateral hamstring autograft
7	26.0	1.68	71	1	Ispilateral hamstring autograft
8	21.5	1.84	75	1	Ispilateral hamstring autograft
9	42.8	1.77	82	1	Ispilateral hamstring autograft

Abbreviation: ACLR, anterior cruciate ligament reconstruction.

The mean PTS decreased by 8.1° from 17.4° (95% CI = [16.6–18.2], ICC = 0.911 [0.680–0.979]) preoperatively to 9.3° (95% CI = [7.2–11.3], ICC = 0.954 [0.257–0.992]) post‐operatively (*p* < 0.001, the *p* value refers to the reduction in PTS from pre‐ to post‐operative). The mean ATT decreased by 7.8 mm from 13.6 mm (95% CI = [10.8–16.4], ICC = 0.904 [0.656–0.977]) preoperatively to 5.8 mm (95% CI = [3.3–8.4], ICC = 0.975 [0.896–0.994]) post‐operatively (*p* < 0.01, the *p* value refers to the reduction in ATT in the operated knee from pre‐ to post‐operative). The side‐to‐side ATT difference decreased from 5.5 mm (95% CI = [3.5–7.5]) preoperatively to −3.1 mm (95% CI = [−6.4 to 0.3]) post‐operatively (*p* < 0.001, the *p* value refers to the reduction in ATT difference between the operated and the non‐operated knee pre‐ to post‐operative). Patient‐specific data for these measurements are listed in Table 2.

**Table 2:** Radiographic measurements of posterior tibial slope (PTS) and anterior tibial translation (ATT) before and after surgery.

	PTS (°)		Side‐to‐side ATT difference (mm)	
Patient	Preoperative	Post‐operative	Preoperative	Post‐operative
1	15.1	7.3	8.5	2.0
2	16.3	7.5	6.8	−5.2
3	17.6	9.3	2.9	−5.4
4	16.7	9.0	7.8	3.1
5	15.7	7.4	4.8	−7.7
6	15.2	5.0	8.8	−7.6
7	17.6	11.9	1.6	−7.1
8	18.9	11.9	5.4	0.5
9	18.5	14.7	3.0	0.0

The reported grades from all clinical stability tests, including Lachman, anterior drawer, and pivot shift tests, were significantly reduced from preoperative grades to post‐operative grades (*p *< 0.001 for all). The tests, preoperative grades and post‐operative grades are all listed in Table 3. The post‐operative IKDC score was 79.4 (range: 60.9–95.4).

**Table 3:** Clinical stability tests for the Lachman, anterior drawer and pivot shift tests (*n*).

	Preoperative	Post‐operative	*p*
Lachman grade			<0.001
0	0	9	
1	0	0	
2	7	0	
3	2	0	
Anterior drawer grade			<0.001
0	0	9	
1	2	0	
2	7	0	
3	0	0	
Pivot shift grade			<0.001
0	0	9	
1	0	0	
2	2	0	
3	7	0	

Regarding adverse events, no infection, deep venous thrombosis or post‐operative hospitalization related to the surgery was found in these patients. One secondary surgery was performed, where hardware removal of the tibial staples was performed due to irritation.

## DISCUSSION

The most important finding of this study was that a revision ACLR using a BPTB autograft combined with an ACWPTO and LET as a single‐stage surgery was a safe and reliable procedure with good clinical outcomes. Treatment of a complex ACL deficiency with anterolateral rotatory laxity and increased PTS and ATT is difficult and has previously been reported using staged surgery or with primarily soft tissue autograft options to lower surgical risks while possibly compromising optimal treatment in reducing the risk of revision ACLR failure.

Regarding the technique for the osteotomy, ACWPTO can be performed proximal to, at the level of or distal to the tibial tubercle. The supratubercle approach has several advantages. First, the osteotomy is performed in pure cancellous bone, decreasing the risk of non‐union. Second, the extensor mechanism compresses the osteotomy site, resulting in the possibility to use two simple staples as fixation and lower the hardware irritation risk and rate of secondary surgery for hardware removal needed. Furthermore, having a high rate of hardware removal counteracts the purpose of performing this surgery in one stage [34]. Finally, since there is no need for a tibial tuberosity osteotomy to access the most proximal tibia, we also retain the possibility of harvesting the BPTB for the revision ACLR graft [30]. It has been reported that the supratubercle approach has risks of patella alta and that the small proximal tibial fragment can compromise fixation of the osteotomy. Recent studies by Tollefson et al. [33] and Demey et al. [11] report that patellar height at 6 months post‐operative was not significantly different from preoperative values, and 99% of patients have sufficient proximal tibial bone to perform a supratubercle closing wedge osteotomy, respectively. Furthermore, risks like non‐optimal tunnel length and placement for the ACL tibial tunnel and iatrogenic damage to the patellar tendon have been proposed; however, this was not our experience during this case series, with no ruptures of the patellar tendon and good clinical stability outcomes.

The optimal graft choice for these patients is still debated, but taking the full scope of current literature into account and consensus from leading experts around the world, BPTB is the preferred graft when lowering the risk of revision ACLR failure is the main goal [21, 29]. Previous studies have reported higher failure rates from hamstring autografts compared to BPTB autografts [23, 24, 36]. Quadriceps grafts are gaining popularity and based on recent research are at least equivalent to hamstring autografts and are comparable to BPTB autografts; however, there is not yet sufficient evidence to suggest a quadriceps graft over a BPTB graft [5, 22, 24, 38, 39]. Autografts, especially in younger patients, are preferred to allografts to increase the potential for graft integration, especially in revision cases [7, 20]. However, in patients with anterior knee pain or patella baja, who might be less suitable for a BPTB harvest due to the risk of decreased patellar height [16] or increased anterior knee pain, a quadriceps tendon autograft or contralateral BPTB autograft could be used with a similar technique. The addition of an LET in patients with a high‐grade pivot shift is also regarded as the current standard.

The results of the objective radiographic measurements and subjective functional outcome match up with previously reported data for patients with supratubercle closing wedge osteotomies and ACL reconstructions. A systematic review by Tollefson et al. [34] reported on studies performing closing wedge osteotomies in the setting of ACL deficiency and reported a range of average decrease in ATT of 4 to 11.5 mm and a range of average decrease in PTS of 0.6° to 11.2°. In that systematic review, 0.6° was an outlier, and most decreases in PTS between the studies were between 6° and 10°. The average decrease in ATT of 8.3 mm reported in this study lines up with the previous study. The average decrease in PTS of 7.5° reported in this study was comparable to previous studies; however, the reason that the final PTS is relatively elevated compared to other studies is that this study reports on a Middle Eastern population, which has a higher baseline PTS than the average population [34, 37].

Several studies have reported that decreases in PTS lead to decreased ATT [1]. In addition, biomechanical studies have reported that decreased PTS leads to decreased ACLR graft force, both of which theoretically lead to decreased ACL graft failures [4]. Furthermore, while it has been reported clinically that LET leads to decreased internal rotation, it is theorized that ACWPTO and LET act synergistically to improve ACLR revision outcomes.

Also, the IKDC score is at the same level as previously reported data from Sonnery‐Cottet et al. [31], Dejour et al. [9] and Rozinthe et al. [27], demonstrating this procedure to be safe and at the very least at the same functional level as the other slightly less technically demanding procedures.

This study has some limitations. This study includes a low number of patients and is not blinded, which could introduce bias, and a larger cohort may affect the results. The study has a retrospective design, and it would be advantageous to have a prospective study to evaluate this surgical technique. Additionally, the moderate follow‐up period could be extended, and further subjective outcome measures could be assessed to provide a more comprehensive assessment of patient‐reported outcomes and potential complications or failure. However, the main findings from this study were looking at radiographic outcomes in which a 1‐year follow‐up is sufficient, and the longest follow‐up for a single patient in this study was more than 6 years, with an excellent outcome, no secondary surgery, and he returned to his desired level of sports. Anecdotally, seven out of nine patients returned to their desired level of sports, while the remaining two patients returned to sports but at a lower level. The lack of additional patient‐reported outcomes is also a limitation because more data would likely provide greater insight into the outcomes and success of surgery for these patients. Furthermore, the lack of a control group, both in the same setting and in a setting without LET or ACWPTO, limits the ability to make a direct comparison, and since it is a combined procedure of osteotomy, revision ACLR, and LET, it is not possible to isolate the effects of the different parts. Future studies should consider using control groups to get further insight.

## CONCLUSION

Single‐stage revision ACLR using a BPTB autograft, LET and ACWPTO in an ACL‐deficient knee with a high‐grade pivot shift and increased PTS was safe and yielded improved clinical outcomes in a small sample size.

## AUTHOR CONTRIBUTIONS

*Substantial contributions to the conception*: Jesper Fritz, Luke V. Tollefson, Robert F. LaPrade and Charles Brown. *Design of the work*: Jesper Fritz, Alan Getgood, Robert F. LaPrade and Charles Brown. *Acquisition, analysis or interpretation of data*: Jesper Fritz, Alan Getgood, Ronald van Heerwaarden, Sebastien Parratte and Charles Brown. *Drafted the work or substantively revised it*: Jesper Fritz, Luke V. Tollefson and Robert F. LaPrade. All authors have approved the submitted version and are accountable for the final version of the manuscript.

## CONFLICT OF INTEREST STATEMENT

Jesper Fritz and Charles Brown are consultants for Smith and Nephew. Robert F. LaPrade is a consultant for Ossur and Smith and Nephew, receives royalties from Ossur, Smith and Nephew and Elsevier, and receives research grants from AANA, AOSSM, Ossur, Arthrex and Smith and Nephew. Alan Getgood is a consultant for Ossur and Smith and Nephew and has equity in Personalized Surgery, Kyniska Robotics, Precision OS and Spring Loaded Technology. Sebastien Parratte is a consultant for Zimmer Biomet Holdings and receives royalties from NewClip Technics and the Rosa Knee System. The remaining authors declare no conflicts of interest.

## ETHICS STATEMENT

All patients provided informed consent for the use of their data for research. Institutional review board approval was obtained for this study (MF3867‐2024‐1).

## Data Availability

The data sets used and analyzed during the current study are available from the corresponding author on reasonable request.
